# Acute Ischemic Stroke Due to Spontaneous Internal Carotid Artery Dissection in a Human Leukocyte Antigen (HLA) B27 Young Male

**DOI:** 10.7759/cureus.22963

**Published:** 2022-03-08

**Authors:** Muhammad Rezeul Huq, Humayun Kabir, Md. Ismail Chowdhury, Ghulam Kawnayn

**Affiliations:** 1 Neurology, Combined Military Hospital, Dhaka, BGD

**Keywords:** hla b27 association, young-onset stroke, hla b27, cervical artery dissection, internal carotid artery dissection

## Abstract

Carotid artery dissection is a significant cause of stroke in young patients. Here, we report a 33-year-old male who presented with right homonymous hemianopia and paresthesia of the right side of the body. Magnetic resonance imaging (MRI) of the brain revealed an acute infarct in the left parieto-occipital region. Magnetic resonance angiography (MRA) and duplex ultrasonography (USG) of the neck vessels suggested the left internal carotid artery dissection as the underlying cause. The patient was a known human leukocyte antigen (HLA) B27 and had a history of a previous attack of uveitis. This case report will raise awareness regarding the possible association of HLA B27 with the dissection of neck vessels.

## Introduction

In a patient with less than 50 years, acute stroke is called the young-onset stroke [1]. Artery dissection, especially internal carotid artery dissection (ICAD), is an established cause of young-onset stroke. In arterial dissection, intimal tear leads to penetration and extension of blood into the arterial wall [2]. It may occur in the elderly population also [3].

Neck trauma due to any cause may lead to this condition. However, no definite reasons are found in most cases, labeled as “Spontaneous Carotid Artery Dissection.” Trivial neck injury during coughing, sneezing, neck exercise, chiropractic manipulation may lead to spontaneous ICAD. Hypertension, migraine, infection, underlying diseases involving arterial wall morphology like fibromuscular dysplasia, Marfan's syndrome, homocysteinemia, Ehler's Danlos syndrome are significantly associated with carotid arterial dissection. The patients usually have neck pain, facial pain or headache, ipsilateral Horner's syndrome. A few days later, they may develop ischemic symptoms like transient ischemic attacks (TIA) or stroke [2].

Human leukocyte antigen (HLA) B27 is a major histocompatibility complex (MHC) class 1 molecule, a classic example of an immunogenic explanation of certain diseases. HLA B27 patients are prone to have various diseases, especially uveitis and spondyloarthropathies (SPA) [4]. It may also associate with arterial diseases like aortitis or aortic dissection in SPA patients [4,5]. However, HLA B27 with carotid arterial dissection is almost unheard of. More data are needed to determine whether the HLA B27 predisposes to arterial dissection.

## Case presentation

A 33-year-old male presented to us with sudden onset paresthesia on the right side of the body and visual disturbance for five days. The visual disturbance was limited to the right visual field of both eyes, leading to bumping into different objects or door during walking. He had no weakness of limbs, unconsciousness, dysphagia, dysarthria, or incontinence. On query, he mentioned neck pain on the left side and bouts of coughing a few days before his symptoms. However, he cannot recollect any definite trauma or injury to the neck.

He suffered from right-sided acute anterior uveitis a few years back. He was found to be HLA B27, now asymptomatic, without any ocular or extraocular manifestations. On examination, he had right-sided homonymous hemianopia. Other examination findings were normal.

**Figure 1:**
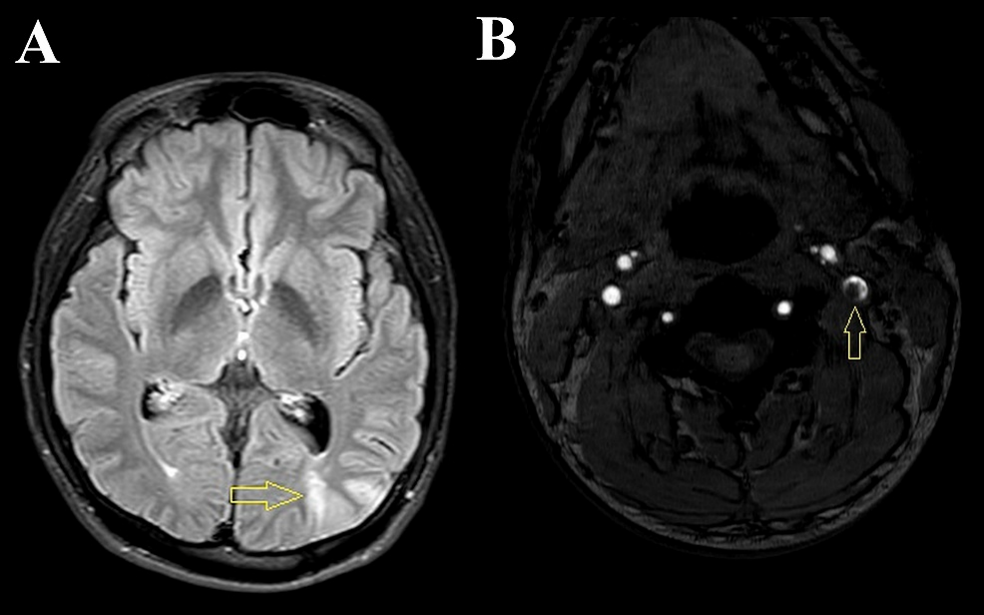
(A) Magnetic resonance imaging (MRI) of the brain fluid-attenuated inversion recovery (FLAIR) sequence showing acute infarct (arrow) in the left parieto-occipital region. (B) The source image of the magnetic resonance angiography (MRA) of the neck shows luminal narrowing due to a thrombus inside the left internal carotid artery (arrow).

**Figure 2:**
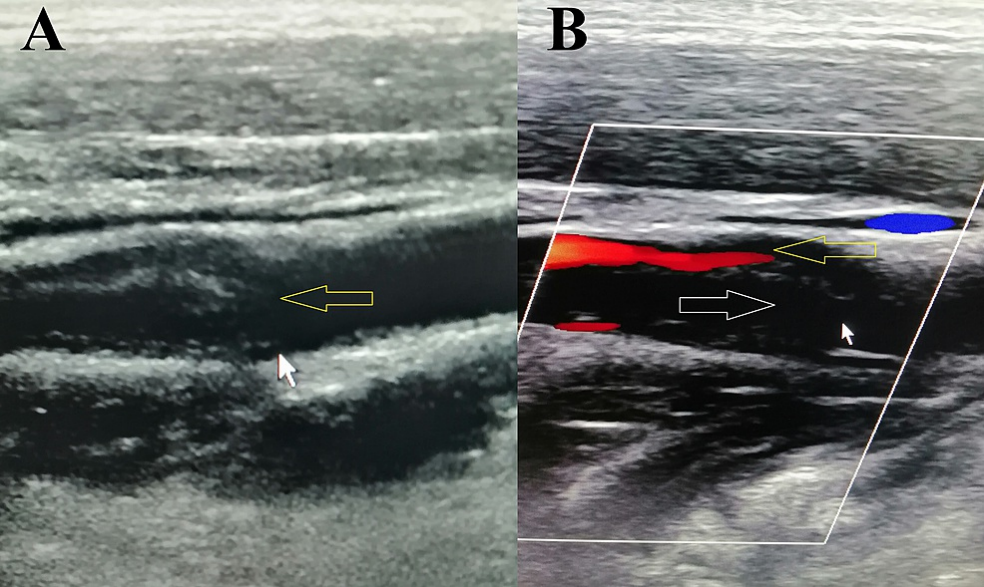
(A) Duplex ultrasonography (B-mode image) shows a dissection flap inside the left internal carotid artery (arrow). (B) Color Doppler image showing narrowed internal carotid artery lumen with blood flow above (yellow arrow) and wide pseudolumen with no blood flow due to thrombus below (white arrow).

**Figure 3:**
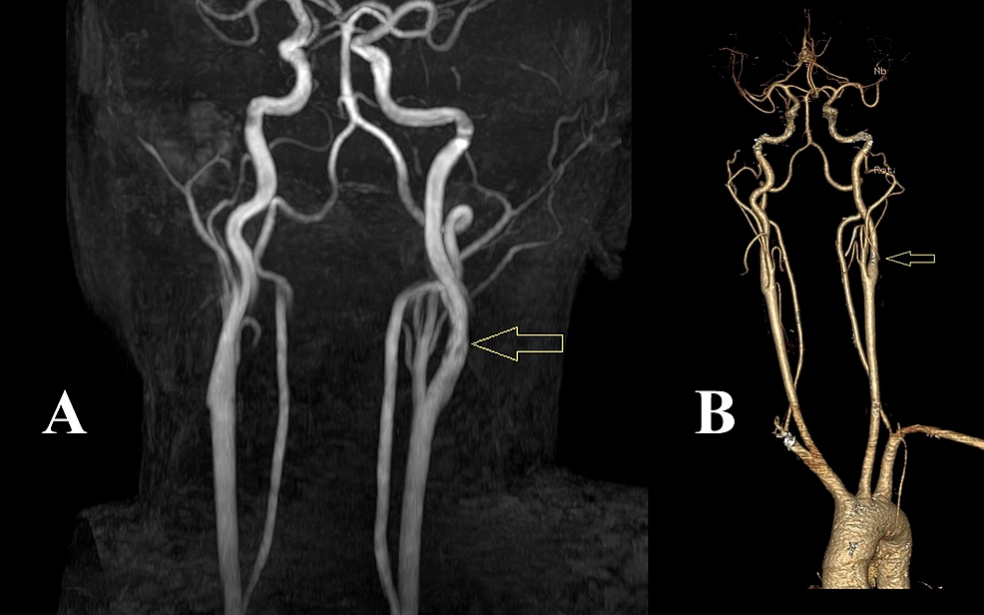
(A) Initial magnetic resonance angiography (MRA) image showing irregular left internal carotid artery wall with intraluminal linear signal voids (arrow) suggesting internal carotid artery dissection. (B) After four weeks, a follow-up computed tomography angiography (CTA) image showed normal architecture of the left internal carotid artery (arrow).

Magnetic resonance imaging (MRI) of the brain revealed acute infarct in the left parieto-occipital region (Figure 1A). We evaluated risk factors, and a duplex study of neck vessels showed the dissection flap in the left internal carotid artery (ICA) at the carotid bulb with the formation of pseudolumen with thrombosis (Figures 2A, 2B). The source image of the magnetic resonance angiography (MRA) of the neck also revealed the thrombus in the left ICA (Figure 1B). The reconstructed MRA image of the neck vessels also showed an irregular lumen of the left ICA with intraluminal thrombus (Figure 3A). Other investigations revealed no abnormalities, including blood sugar, lipid profile, autoimmune profile, Electrocardiography (ECG), Echocardiography, and hypercoagulability workup.

We treated him with enoxaparin 60 mg twice daily for seven days, followed by dabigatran 110 mg twice daily for one month. Before discharge, we did a computed tomography angiography (CTA) of the neck vessels to exclude any flow restriction in the left ICA (Figure 3B). As the CTA was unremarkable, we stopped anticoagulants after one month.

We discharged the patient after three weeks, and he was asymptomatic except for the visual field defect. He also had no new complaints when he came for follow-up after three months.

## Discussion

Though there is no universal definition, most authors recognize stroke before 50 years of age as young-onset stroke. This group of stroke has different etiologies in comparison to the older population. ICAD is responsible for about 10%-25% of ischemic stroke cases in young patients [1].

During arterial dissection, a thrombus develops in the arterial wall. Blood flow may be compromised due to hemodynamic alteration or embolism leading to TIA or stroke. However, these features are usually preceded by headache or neck pain. Horner's syndrome and pain with focal cerebral ischemia form the classic triad of clinical features. The patients may also have syncope, retinal ischemia, or cranial nerve palsies. In some cases, there will be no symptoms. Our patient presented with unilateral headache and neck pain initially, and about after seven days, he developed a sudden onset visual field defect with hemisensory disturbance [2].

ICAD can be diagnosed by vascular imaging in any form like Duplex ultrasonography (USG), MRA, CTA, or digital subtraction angiography (DSA). As a simple, non-invasive procedure, we initially did a duplex study of neck vessels, which revealed a dissection flap and thrombus in the left ICA. Features of carotid stenosis, occlusion, or high resistance flow can also be found in the duplex scan of neck vessels. Combining transcranial Doppler (TCD) with duplex USG of extracranial carotid vessels increases the chance of diagnosing the condition [2,6].

MRI of the neck and MRA of the neck vessels also has high sensitivity and specificity in detecting ICAD. MRI of the neck usually reveals the thrombus in the arterial wall creating pseudolumen and crescent signs. Long segment narrowing, string sign, or a double-lumen of ICA can be seen in MRA of neck vessel. The irregular arterial wall also gives a clue for possible dissection. CTA also offers a similar picture. In our case, we found an irregular vessel wall with an irregular linear filling defect suggesting dissection with thrombus in the proximal segment of the left ICA. However, DSA remains the gold standard test to detect ICAD [2,6]. We did not go for an invasive procedure like DSA as the diagnosis was already established, and there was no indication for neurointervention in our case.

As there was no definite history of trauma, we labeled our case as a spontaneous ICAD. We did not find any other possible association of ICAD in our patient. However, our patient was HLA B27, and we know HLA B27 is associated with many diseases, especially SPA and uveitis. Our patient had a previous episode of acute anterior uveitis, which recovered completely. Other arterial diseases, specially aortitis, and aortic dissection were reported previously in HLA B27 ankylosing spondylitis (AS) patients. Spontaneous aortic dissection was reported in AS patients, but the relationship between HLA B27 and aortic dissection is still debated [4,5]. HLA B27 negative AS patients may also have aortic dissection [7]. We found a case report of an HLA B27 child with vertebral artery dissection, which again raises the question of a possible association of HLA B27 with arterial dissections [8].

How HLA B27 causes arterial diseases is not established yet. It is thought that inflammatory reactions leading to vessel wall changes are responsible for developing aortic and aortic valve diseases in HLA B27 AS patients [9]. Similar mechanisms may be responsible for the development of carotid arterial disease in HLA B27 patients.

The treatment of ICAD is primarily conservative. Though no uniform guidelines are available, anticoagulants are given conventionally with good results. Either heparin or oral anticoagulants can be used. Usually, more than three months of treatment are given [10]. However, as the follow-up CTA in our patient was completely normal, we discontinued the anticoagulant after one month.

## Conclusions

ICAD is an important cause of ischemic stroke, especially in young patients. We reported this young male with ischemic stroke due to ICAD with no other association except HLA B27. It is too early to say whether carotid or other arterial dissections will be recognized as HLA B27 associated diseases. HLA B27 could be tested in patients with ICAD to see the actual scenario. Also, in HLA B27 patients with ischemic stroke, we should exclude carotid or vertebral artery dissection as a possible underlying cause.
